# Supplementary material for: Neutrophil extracellular traps promote immunopathogenesis of virus-induced COPD exacerbations
Source: Nat Commun. 2024 Jul 9;15:5766. doi: 10.1038/s41467-024-50197-0 (PMC11233599; doi:10.1038/s41467-024-50197-0)
Supplement: Supplementary file 1 — Supplementary Information [file 41467_2024_50197_MOESM1_ESM.pdf]

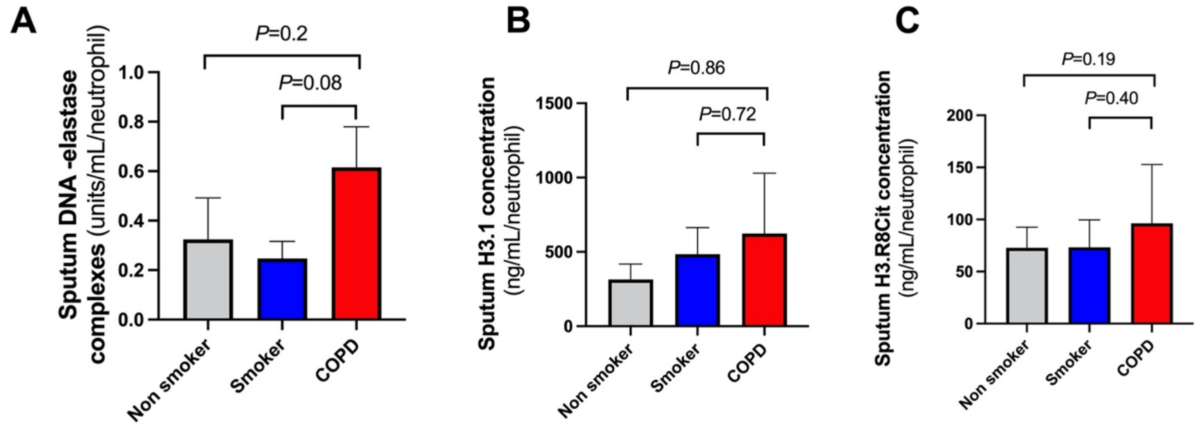

Supplementary Figure 1: Sputum concentrations of NETosis markers adjusted for total neutrophil counts in experimentally infected subjects. Measurement of (A) DNA-elastase (n=8 non smoker, n=10 smoker, n=9 COPD), (B) H3.1 (n=9 non-smoker, n= 10 smoker, n=9 COPD) and (C) H3.R8Cit (n=9 non-smoker, n= 10 smoker, n=9 COPD) in sputum at day 9 post-infection divided by sputum neutrophil counts at the same timepoint. Data are presented as mean values +/- SEM and analysed by two-tailed Mann Whitney U test. Source data are provided as a Source Data file.

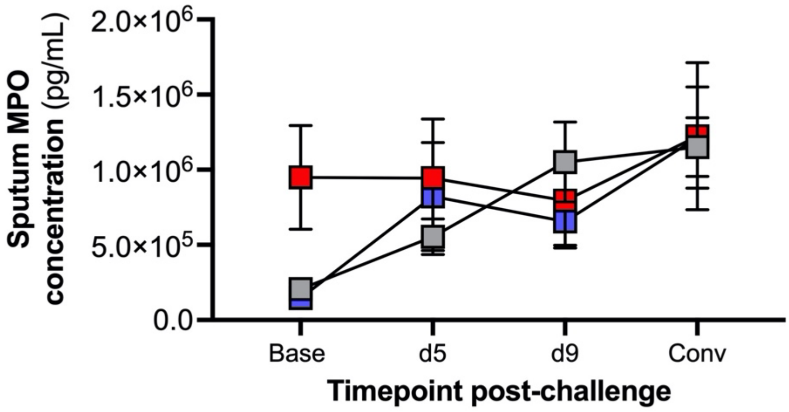

Supplementary Figure 2: Sputum myeloperoxidase concentrations during human RV infection in COPD. Sputum myeloperoxidase (MPO) was quantified by ELISA subjects with COPD (n=9), healthy smokers (n=10) and healthy non-smokers (n=10) following experimental RV infection. Data are presented as mean values +/- SEM. Source data are provided as a Source Data file.

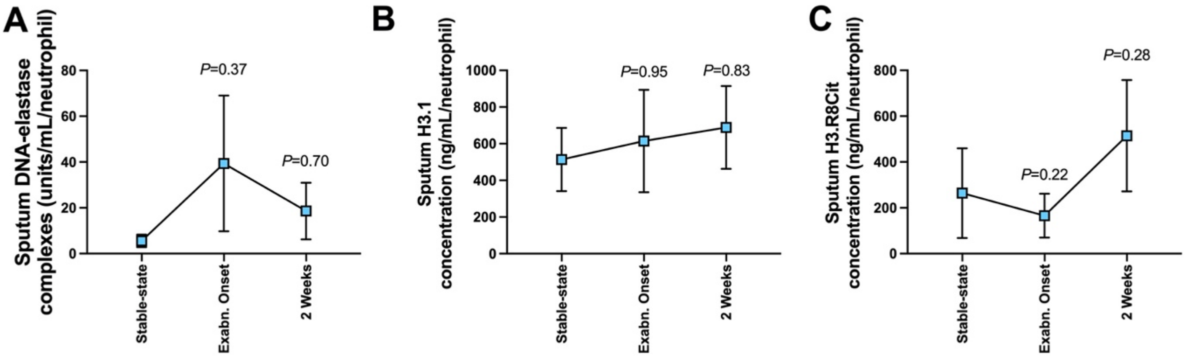

Supplementary Figure 3: Sputum concentrations of total histones and NETosis markers adjusted for total neutrophil counts in naturally occurring COPD exacerbations. Measurement of (A) DNA/elastase complexes (B) total intact (H3.1) and (C) citrullinated (H3.R8) nucleosomes by ELISA in sputum at stable-state, exacerbation onset and 2 weeks post-onset, divided by sputum neutrophil counts at the same timepoint. Data analysed by two-tailed Wilcoxon matched pairs, signed-rank test to compare steady-state with exacerbation onset or 2 weeks after exacerbation. Source data are provided as a Source Data file.

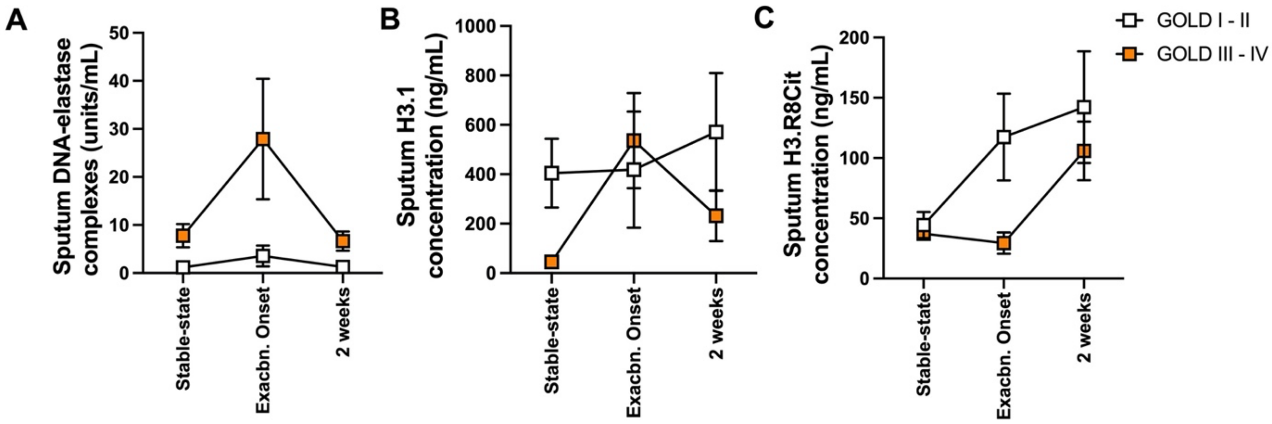

Supplementary Figure 4: Airway NET expression during naturally occurring virus associated exacerbations in COPD subjects stratified according to GOLD severity class.

Sputum concentrations of (A) DNA-elastase complexes, (B) total intact (H3.1) and (C) citrullinated (H3.R8) nucleosomes were measured at stable-state and following naturally occurring virus-induced exacerbation. Data are presented as mean values +/- SEM and analysed by two-tailed Wilcoxon matched pairs, signed-rank test to compare steady-state with exacerbation onset or 2 weeks after exacerbation. Source data are provided as a Source Data file.

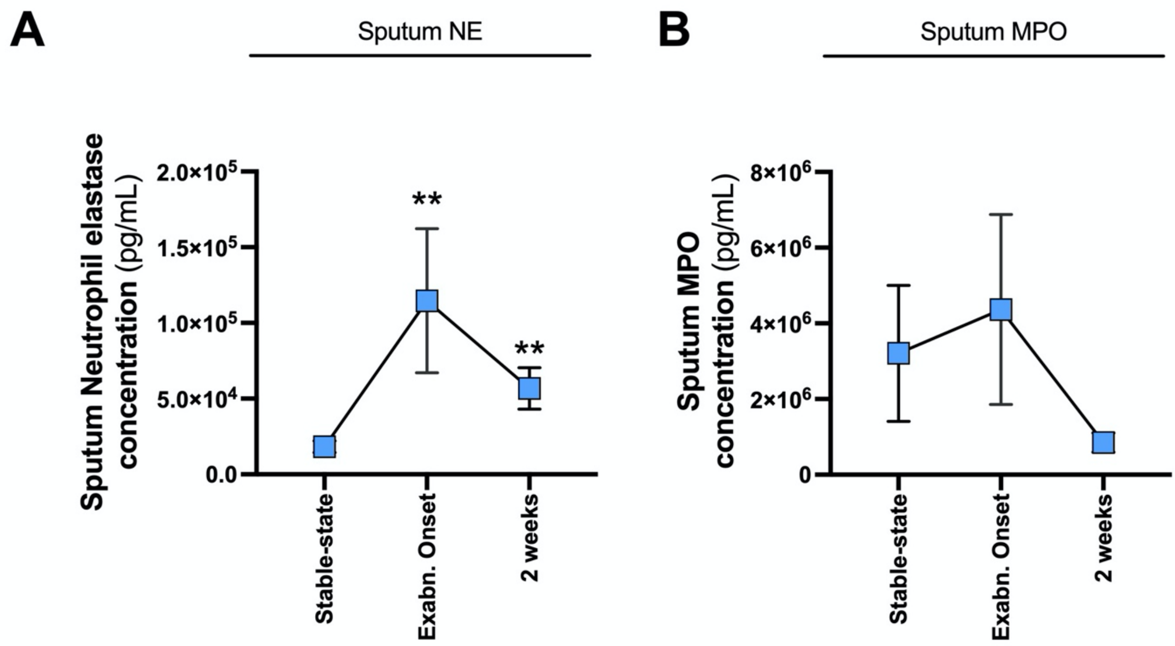

Supplementary Figure 5: Sputum neutrophil elastase and myeloperoxidase during naturally occurring virus associated exacerbations. Sputum concentrations of (A) Neutrophil elastase (NE) and (B) myeloperoxidase (MPO) were measured at stable-state and following naturally occurring virus-induced exacerbation. Data are presented as mean values +/- SEM. and analysed by two-tailed Wilcoxon matched pairs, signed-rank test to compare steady-state with exacerbation onset or 2 weeks after exacerbation. ***P*<0.01. Source data are provided as a Source Data file.

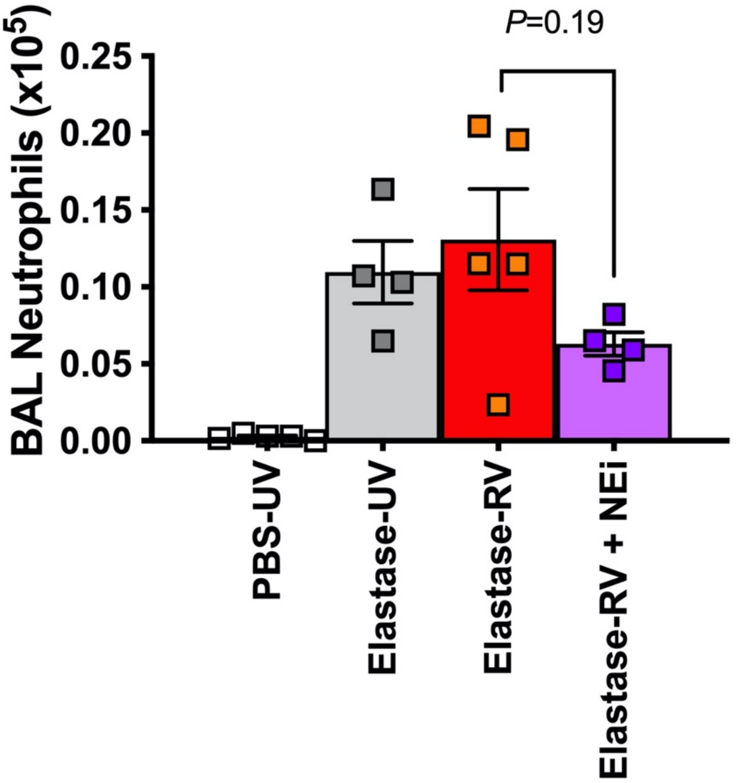

Supplementary Figure 6: Effect of pharmacological neutrophil elastase inhibition upon neutrophil recruitment at day 4 post RV infection. BAL neutrophils were enumerated by cytospin in mice treated with elastase 10 days prior to infection with RV-A1 treated with Neutrophil elastase inhibitor or vehicle control. n=4-5 mice/group, representative of at least two independent experiments. Data presented as mean+/-SEM. Data analysed by two-tailed Mann Whitney U test. Source data are provided as a Source Data file.

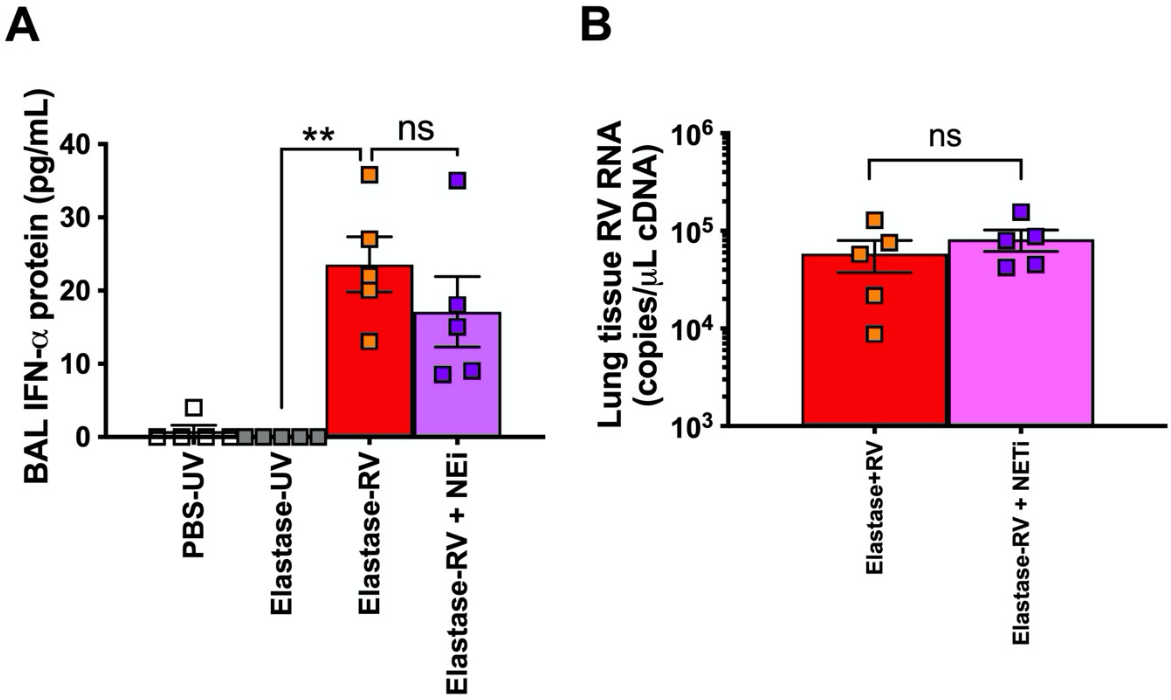

Supplementary Figure 7: Pharmacological neutrophil elastase inhibition has no impact upon antiviral immunity. Mice were treated with elastase 10 days prior to treatment with Neutrophil elastase inhibitor or vehicle control and infection with RV-A1. (A) Bronchoalveolar lavage (BAL) concentrations of IFN-α were quantified by ELISA. (B) Lung tissue rhinovirus RNA copies were quantified by qPCR. n=5 mice/group, representative of at least two independent experiments. Data presented as mean+/-SEM. Data analysed by two-tailed Mann Whitney U test. ***P*<0.01, ns =non-significant. Source data are provided as a Source Data file.

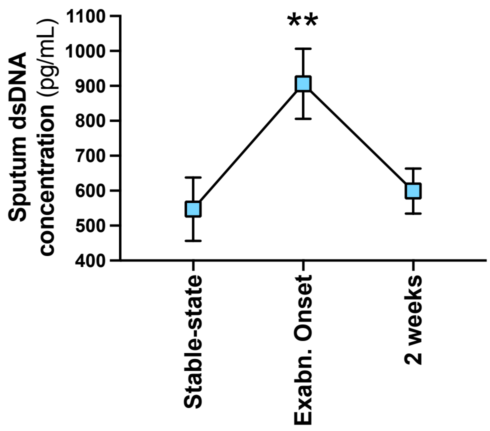

Supplementary Fig 8: Airway dsDNA expression during naturally occurring, virus-induced COPD exacerbations. Sputum concentrations of dsDNA were measured at stable-state and following naturally occurring virus-induced exacerbation. ***P*<0.01. Data analysed by Wilcoxon matched pairs, signed-rank test Data are presented as mean values +/- SEM. and analysed by two-tailed Wilcoxon matched pairs, signed-rank test to compare steady-state with exacerbation onset or 2 weeks after exacerbation. ***P*<0.01. Source data are provided as a Source Data file.

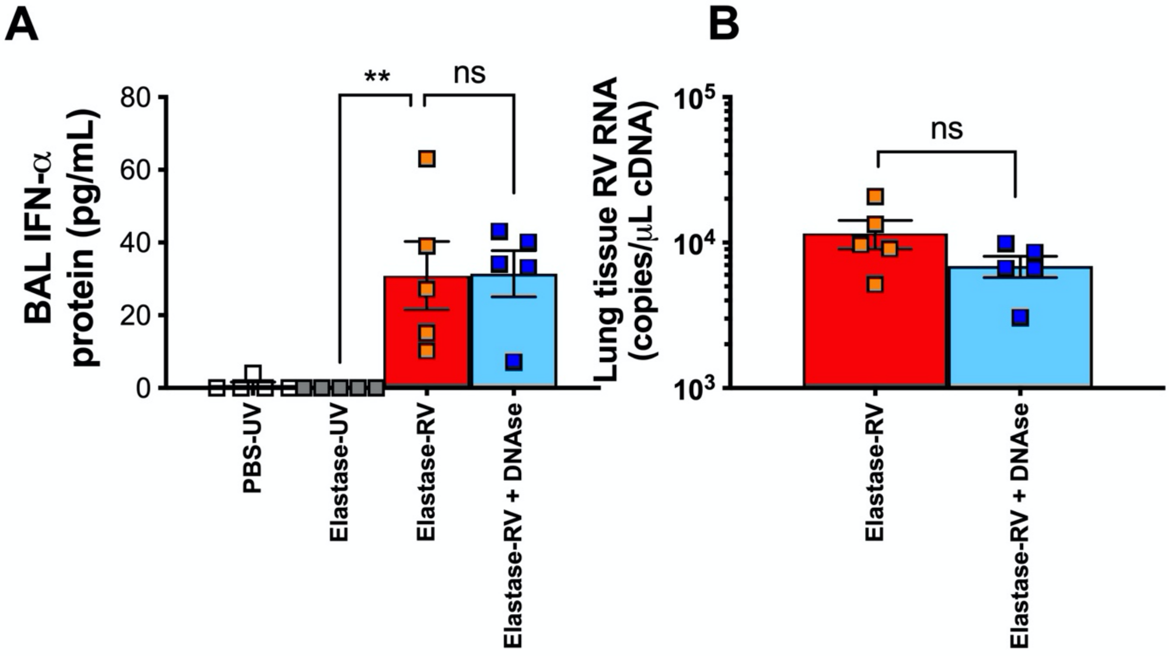

Supplementary Figure 9: DNAse treatment has no impact upon antiviral immunity. Mice were treated with elastase 10 days prior to treatment with DNAse or vehicle control and infection with RV-A1. (A) Bronchoalveolar lavage (BAL) concentrations of IFN-α were quantified by ELISA. (B) Lung tissue rhinovirus RNA copies were quantified by qPCR. n=5 mice/group, representative of at least two independent experiments. Data presented as mean+/-SEM and analysed by two-tailed Mann Whitney U test. ***P*<0.01, ns =non-significant. Source data are provided as a Source Data file.

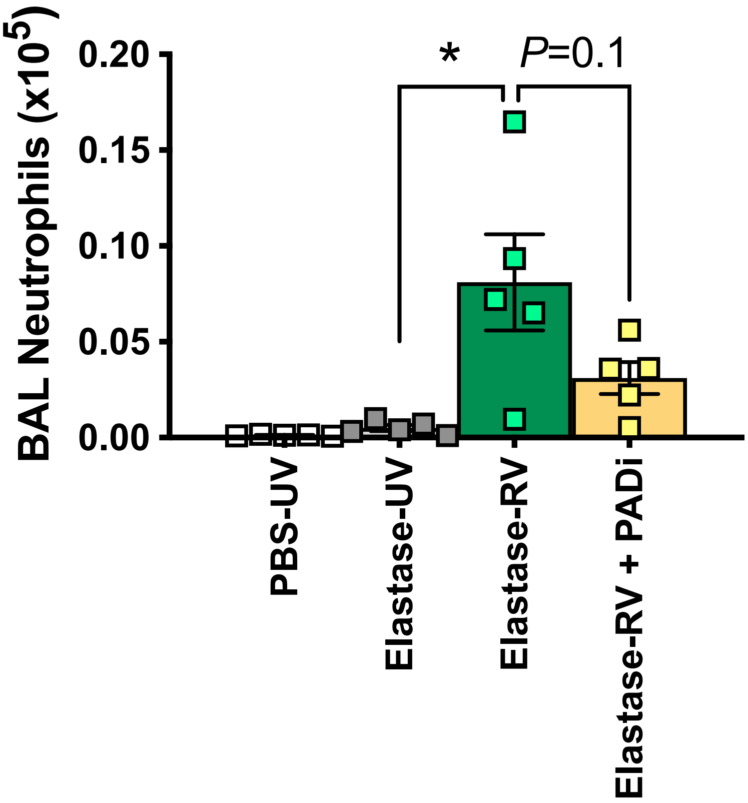

Supplementary Figure 10: Effect of pharmacological NET inhibition upon neutrophil recruitment at day 4 post RV infection. BAL neutrophils were enumerated by cytospin in mice treated with elastase 10 days prior to infection with RV-A1 treated with PAD inhibitor (BB-CL-amidine) or vehicle control. n=5 mice/group, representative of at least two independent experiments. Data presented as mean+/-SEM and analysed by two-tailed Mann Whitney U test. $^*P<0.05$. Source data are provided as a Source Data file.

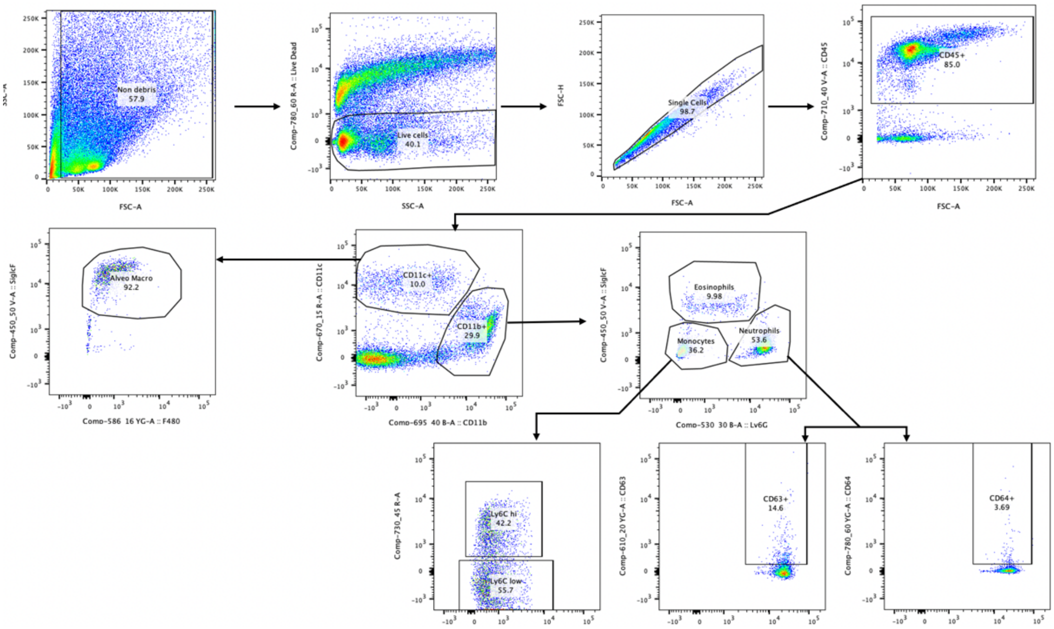

Supplementary Figure 11: Flow cytometry Gating strategy for identification of neutrophils in mouse samples
