# Supplementary material for: Neutrophil extracellular traps promote immunopathogenesis of virus-induced COPD exacerbations
Source: Nat Commun. 2024 Jul 9;15:5766. doi: 10.1038/s41467-024-50197-0 (PMC11233599; doi:10.1038/s41467-024-50197-0)
Supplement: Supplementary file 3 — Reporting Summary [file 41467_2024_50197_MOESM3_ESM.pdf]

nature portfolio

Corresponding author(s): Aran Singanayagam

Last updated by author(s): 23/05/24

Reporting Summary

Nature Portfolio wishes to improve the reproducibility of the work that we publish. This form provides structure for consistency and transparency in reporting. For further information on Nature Portfolio policies, see our Editorial Policies and the Editorial Policy Checklist.

Statistics

For all statistical analyses, confirm that the following items are present in the figure legend, table legend, main text, or Methods section.

n/a	Confirmed	
☐	☒	The exact sample size (*n*) for each experimental group/condition, given as a discrete number and unit of measurement
☐	☒	A statement on whether measurements were taken from distinct samples or whether the same sample was measured repeatedly
☐	☒	The statistical test(s) used AND whether they are one- or two-sided<br>*Only common tests should be described solely by name; describe more complex techniques in the Methods section.*
☒	☐	A description of all covariates tested
☒	☐	A description of any assumptions or corrections, such as tests of normality and adjustment for multiple comparisons
☐	☒	A full description of the statistical parameters including central tendency (e.g. means) or other basic estimates (e.g. regression coefficient) AND variation (e.g. standard deviation) or associated estimates of uncertainty (e.g. confidence intervals)
☒	☐	For null hypothesis testing, the test statistic (e.g. *F*, *t*, *r*) with confidence intervals, effect sizes, degrees of freedom and *P* value noted<br>*Give P values as exact values whenever suitable.*
☒	☐	For Bayesian analysis, information on the choice of priors and Markov chain Monte Carlo settings
☒	☐	For hierarchical and complex designs, identification of the appropriate level for tests and full reporting of outcomes
☒	☐	Estimates of effect sizes (e.g. Cohen's *d*, Pearson's *r*), indicating how they were calculated

Our web collection on statistics for biologists contains articles on many of the points above.

Software and code

Policy information about availability of computer code

Data collection	No software used
Data analysis	GraphPad Prism 10 (GraphPad Software)

For manuscripts utilizing custom algorithms or software that are central to the research but not yet described in published literature, software must be made available to editors and reviewers. We strongly encourage code deposition in a community repository (e.g. GitHub). See the Nature Portfolio guidelines for submitting code & software for further information.

Data

Policy information about availability of data

All manuscripts must include a data availability statement. This statement should provide the following information, where applicable:

- Accession codes, unique identifiers, or web links for publicly available datasets
- A description of any restrictions on data availability
- For clinical datasets or third party data, please ensure that the statement adheres to our policy

The data supporting the findings of the study are available in this article and its supplementary Information files, or from the corresponding author on request. Source data are provided with this paper.

Research involving human participants, their data, or biological material

Policy information about studies with human participants or human data. See also policy information about sex, gender (identity/presentation), and sexual orientation and race, ethnicity and racism.

Reporting on sex and gender	Data from human participants in the human exacerbation studies derives from male and female sex individuals. There was a non-significant trend towards a greater % males in the COPD group which is in keeping with the fact that the disease more commonly affects males. These data are shown in Table 1.
Reporting on race, ethnicity, or other socially relevant groupings	n/a
Population characteristics	Age, smoking history, disease severity and treatment use are reported in Tables 1 and 2
Recruitment	Subjects were recruited through advertisements in local newspapers and on the internet. Attempts to recruit through hospital outpatient clinics, general practitioners surgeries or support groups were largely unsuccessful, mainly due to the disease severity of COPD patients in these settings. Initial contact with volunteers was made by telephone and a pro forma screening questionnaire completed, this enabled exclusion of unsuitable subjects and the formation a database for volunteers interested in future research projects. In total approximately 1550 subjects were screened by telephone. Of those 176 attended hospital clinic in person for full screening. Approximately 45% of subjects were subsequently excluded after being found to be RV16 seropositive.
Ethics oversight	St Mary's NHS Trust Research Ethics Committee (study number 07/H0712/138) and East London Research Ethics Committee (study number 11/LO/0229). Informed consent was obtained from all participants.

Note that full information on the approval of the study protocol must also be provided in the manuscript.

Field-specific reporting

Please select the one below that is the best fit for your research. If you are not sure, read the appropriate sections before making your selection.

☒ Life sciences ☐ Behavioural & social sciences ☐ Ecological, evolutionary & environmental sciences

For a reference copy of the document with all sections, see nature.com/documents/nr-reporting-summary-flat.pdf

Life sciences study design

All studies must disclose on these points even when the disclosure is negative.

Sample size	This was an exploratory study and we were unable to perform a power calculation as this is the first study of its kind. Given the resources available for the study, ~10 study subjects per arm was also a practical and achievable number.
Data exclusions	Nil
Replication	Independent replication in a validation human cohort was not feasible for this study. All animal studies were reproduced in two independent experiments
Randomization	Experimental groups were defined by presence or absence of COPD and therefore randomisation not possible/relevant
Blinding	All experimental assays were performed with the researchers blind to the treatment received

Reporting for specific materials, systems and methods

We require information from authors about some types of materials, experimental systems and methods used in many studies. Here, indicate whether each material, system or method listed is relevant to your study. If you are not sure if a list item applies to your research, read the appropriate section before selecting a response.

Materials & experimental systems

n/a	Involved in the study
	[x] Antibodies
[x]	Eukaryotic cell lines
[x]	Palaeontology and archaeology
	[x] Animals and other organisms
	[x] Clinical data
[x]	Dual use research of concern
[x]	Plants

Methods

n/a	Involved in the study
[x]	ChIP-seq
	[x] Flow cytometry
[x]	MRI-based neuroimaging

Antibodies

Antibodies used	CD45: BV711 clone 30-F11 eBioscience (dilution 1:200)<br>CD11b PerCP clone M1170 eBioscience (dilution 1:300)<br>CD11c APC clone HL-3 BD Pharmigen (dilution 1: 100)<br>F480 PE Clone BM8 eBioscience (dilution 1:100)<br>Ly6G FITC clone 1A8 BD Pharmigen (dilution 1:100)<br>CD63 PEDzl clone NVG-2 Biolegend (dilution 1:100)<br>CD64 PECy7 X54-5/7.1 Biolegend (dilution 1:50)
Validation	Please see manufacturer's websites for relevant details

Animals and other research organisms

Policy information about studies involving animals; ARRIVE guidelines recommended for reporting animal research, and Sex and Gender in Research

Laboratory animals	Female mice (6–8 weeks of age) on a C57/BL6 background housed in individually ventilated cages within specific pathogen free conditions. Mice had access to food and water ad libitum with 12 hour alternating light/dark cycles at temperature of 20-24 degrees celsius. All animals were purchased from Charles River Laboratories UK
Wild animals	No wild animals used in this study
Reporting on sex	Female mice alone were used in our animal experiments. This is in line with our prior work in these models which have typically used females for consistency. Male mice have an increased propensity to fight which can invoke a stress response which may affect data generated.
Field-collected samples	No field samples were collected in this study.
Ethics oversight	All animal work was performed under the authority of the UK Home Office outlined in the Animals (Scientific Procedures) Act 1986 after ethical review by Imperial College London Animal Welfare and Ethical Review Body (project licences 70/7234 and PP4051423).

Note that full information on the approval of the study protocol must also be provided in the manuscript.

Clinical data

Policy information about clinical studies
All manuscripts should comply with the ICMJEguidelines for publication of clinical research and a completedCONSORT checklist must be included with all submissions.

Clinical trial registration	n/a - not a clinical trial
Study protocol	n/a - not a clinical trial
Data collection	Clinical recruitment and data collection occurred from Subjects were recruited January 2008 and December 2010 (human experimental challenge study) and from June 2011 and December 2013 (naturally occurring exacerbation study)
Outcomes	Our primary outcome was quantification of NETs in sputum samples

Plants

Seed stocks	n/a
Novel plant genotypes	n/a
Authentication	n/a

Flow Cytometry

Plots

Confirm that:

- [x] The axis labels state the marker and fluorochrome used (e.g. CD4-FITC).
- [x] The axis scales are clearly visible. Include numbers along axes only for bottom left plot of group (a 'group' is an analysis of identical markers).
- [x] All plots are contour plots with outliers or pseudocolor plots.
- [x] A numerical value for number of cells or percentage (with statistics) is provided.

Methodology

Sample preparation	Cells were stained with the Live/Dead Fixable Near-IR-Dead Cell staining kit (Invitrogen) for 20 minutes in PBS prior to blockade with anti-CD16/CD32 Fc receptor block (BD Pharminogen) for 20 minutes. Cells were then washed in PBS containing 0.1% sodium azide and 1% BSA followed by staining for surface markers at 40C for 30 minutes. Cells were subsequently washed in PBS containing 0.1% sodium azide and 1% BSA before fixation in 2% paraformaldehyde
Instrument	BD LSRFortessa
Software	Collection of Data: BD FACSDiva<br>Analysis of Data: FlowJo
Cell population abundance	n/a - no sorting
Gating strategy	Shown in Supplementary Figure 11

- [x] Tick this box to confirm that a figure exemplifying the gating strategy is provided in the Supplementary Information.
